# CXCR3 mediates ascites-directed tumor cell migration and predicts poor outcome in ovarian cancer patients

**DOI:** 10.1038/oncsis.2017.29

**Published:** 2017-05-15

**Authors:** C Windmüller, D Zech, S Avril, M Boxberg, T Dawidek, B Schmalfeldt, M Schmitt, M Kiechle, H Bronger

**Affiliations:** 1 Department of Gynecology and Obstetrics, Technical University of Munich, Munich, Germany; 2 Department of Pathology, Technical University of Munich, Munich, Germany; 3 Department of Gynecology, University Medical Center Hamburg-Eppendorf, Hamburg, Germany

## Abstract

Intraabdominal tumor dissemination is a major hallmark of epithelial ovarian cancer (EOC), but the underlying mechanisms have not been fully elucidated. The CXCR3 chemokine receptor supports migration of tumor cells to metastatic sites, but its role in ovarian cancer metastasis is largely unknown. Herein, we first screened two independent cohorts of high-grade serous ovarian cancers (HGSCs, discovery set *n*=60, validation set *n*=117) and 102 metastatic lesions for CXCR3 expression. In primary tumors, CXCR3 was particularly overexpressed by tumor cells at the invasive front. In intraabdominal metastases, tumor cells revealed a strong CXCR3 expression regardless of its expression in the corresponding primary tumor, suggesting a selection of CXCR3-overexpressing cancer cells into peritoneal niches. In support of this, CXCR3 mediated the migration of tumor cell lines OVCAR3 and SKOV3 toward malignant ascites, which was inhibited by a monoclonal anti-CXCR3 antibody *in vitro*. These results were prospectively validated in ascites-derived tumor cells from EOC patients *ex vivo* (*n*=9). Moreover, tumor cell-associated overexpression of CXCR3 in advanced ovarian cancer patients was associated with a reduced progression-free survival (PFS) and overall survival (OS), which remained independent of optimal debulking, age, FIGO stage and lymph node involvement (PFS: hazard ratio (HR) 2.11, 95% confidence interval (CI) 1.30–3.45, *P*=0.003; OS: HR 2.36, 95% CI 1.50–3.71, *P*<0.001). These results in ovarian cancer patients identify CXCR3 as a potential new target to confine peritoneal spread in ovarian cancer after primary cytoreductive surgery.

## Introduction

Intraabdominal metastasis remains a fundamental clinical challenge in advanced ovarian cancer and accounts for disease recurrence and death in most of the patients.^1^ Peritoneal spread is generally understood as a process of three consecutive steps: (1) migration of cancer cells out of the primary tumor and detachment from its surface into the abdominal cavity, (2) passive distribution along the physiologic current of the peritoneal fluid and (3) adhesion to the mesothelium leading to peritoneal and omental macrometastases.^1, 2^ Although several candidates, such as matrix metalloproteases or integrins, participate in the last step, the mechanisms used by ovarian cancer cells to escape from the primary tumor remain poorly defined.

Chemokines physiologically facilitate chemotactic migration of leukocytes.^3^ However, cancer cells upregulate chemokine receptors and exploit them to migrate along chemokine gradients to distant metastatic sites.^4^ It thus seems conceivable that chemokine receptors may as well participate in the migration of ovarian cancer cells toward the abdominal cavity. Similar mechanisms have been described for lysophosphatidic acid receptors, the hepatocyte growth factor/cMET axis and for the CXCR4 receptor.^5, 6, 7, 8^

The CXCR3 chemokine receptor is such a conceivable metastasis-promoting factor as it is upregulated in ovarian carcinomas^9, 10^ and involved in cancer growth and metastasis of other solid cancers.^11, 12^ Physiologically, it is predominantly expressed on T lymphocytes and natural killer cells.^13^ Recently, we have shown that high intratumoral concentrations of the two CXCR3 ligands CXCL9 and CXCL10 are associated with improved survival in high-grade serous ovarian cancer (HGSC).^14^ This protective effect of intratumoral CXCR3 chemokines is generally attributed to an enhanced infiltration of CXCR3-positive tumor-suppressive lymphocytes.^15, 16^ However, tumor-promoting regulatory T cells also use CXCR3 to home into ovarian cancers.^14, 17^ We therefore speculated that other mechanisms may be involved in the protective effect of high intratumoral CXCL9 and CXCL10 concentrations, namely the prevention of CXCR3-mediated tumor cell migration out of the primary tumor.

To address this hypothesis in humans, we explored expression, function and prognostic impact of tumor cell CXCR3 in ovarian cancer patients. We demonstrate that CXCR3 is expressed at the invasive front of ovarian carcinomas and overexpressed in intraabdominal metastases. Moreover, it mediates migration of cancer cells toward malignant ascites both in ovarian cancer cell lines *in vitro* and in primary ascites-derived cancer cells from patients *ex vivo*. Finally, we identify and validate CXCR3 as an independent marker of reduced progression-free survival (PFS) and overall survival (OS) in advanced HGSC. These results imply that CXCR3 may contribute to tumor cell dissemination within the abdominal cavity in humans, making it a potential target structure to control peritoneal spread after primary debulking surgery.

## Results

### CXCR3 is overexpressed by tumor cells at the invasive front and in intraabdominal metastases of HGSC

**Figure 1:**
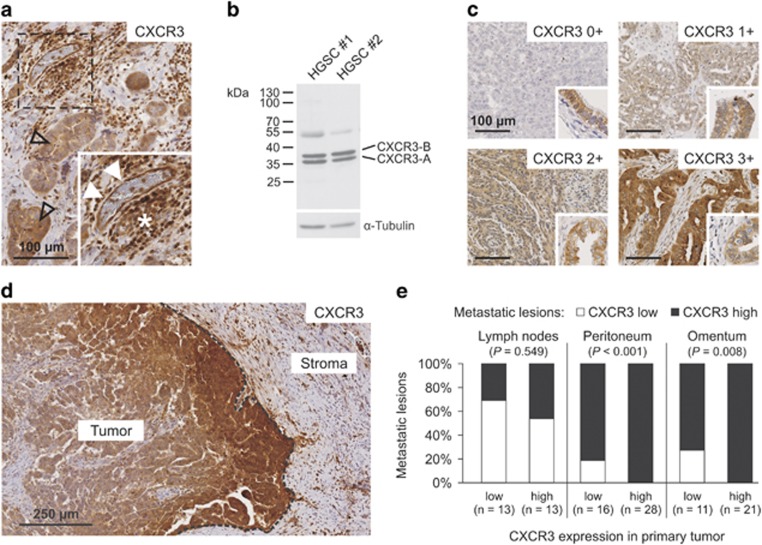
Expression of CXCR3 in HGSCs. (**a**) CXCR3 is predominantly expressed by tumor cells (open arrowheads), endothelial cells (solid arrowheads) and tumor-infiltrating lymphocytes (asterisk). Negative control is shown in Supplementary Figure S3. (**b**) Western blot analysis of tissue extracts from HGSC samples stained with the monoclonal antibody directed to CXCR3 used for immunohistochemistry showed specificity for CXCR3-A and CXCR3-B. The weaker band at 55 kDa is a remnant of the loading control (α-tubulin). (**c**) CXCR3 expression in tumor cells was scored semiquantitatively in a four-tiered grading system. Fallopian tube epithelium served as a positive control (inserts). (**d**) Representative example of a HGSC showing pronounced CXCR3 expression at the invasive front (dashed line) compared with the tumor center. (**e**) Tumor cell CXCR3 expression was scored in 102 HGSC metastases. In contrast to lymph node metastases (*n*=26), tumor cells in peritoneal (*n*=44) or omental metastases (*n*=32) almost exclusively showed strong (2+ or 3+) CXCR3 expression irrespective of its expression in the corresponding primary tumor. *P-*values were calculated using McNemar’s statistical test.

CXCR3 expression was analyzed immunohistochemically in 60 specimens of advanced HGSC (‘discovery set’, Table 1). The receptor was localized in tumor cells, endothelial cells and tumor-infiltrating lymphocytes, whereas stromal fibroblasts were negative (Figure 1a). Western blot analysis of HGSC tissue extracts using the same monoclonal antibody (MAB160) further confirmed its specificity, showing two distinct bands between 35 and 40 kDa representing the two splice variants CXCR3-A and CXCR3-B (Figures 1b, band at 55 kDa is a remnant of the loading control α-tubulin).^18^ In cases of CXCR3 positivity, 100% of tumor cells displayed CXCR3 expression. The expression of CXCR3 in tumor cells was scored semiquantiatively as 0, 1+, 2+ or 3+ (Figure 1c). In the discovery set, tumor cell CXCR3 was scored 0 (2/60, 3%), 1+ (13/60, 22%), 2+ (28/60, 47%) and 3+ (17/60, 28%). Particularly high CXCR3 expression was observed at the invasive front in about one-third of the cases (Figure 1d). Next, we conducted an unbiased survey of the expression of CXCR3 in abdominal lymph node (*n*=26), peritoneal (*n*=44) and omental metastases (*n*=33) and compared it with its expression in the corresponding primary tumors. In lymph nodes, there was no correlation between CXCR3 levels in the primary and the metastatic tumor cells (Figure 1e). However, in both peritoneal and omental metastases we almost exclusively found CXCR3-overexpressing cancer cell populations regardless of its expression in the corresponding primary tumor (immunohistochemistry score 2+ or 3+, Figure 1e). Using McNemar’s statistical test, this shift toward a high CXCR3 expression in intraabdominal metastases was highly significant (*P*<0.001 and *P*=0.008 for peritoneal and omental metastases, respectively). Based on these results, we speculated that the CXCR3 chemokine receptor may be involved in the initial step of peritoneal metastasis through promoting migration of cancer cells from the primary tumor toward the peritoneal cavity.

### CXCR3 is functionally active in EOC cells and mediates tumor cell migration toward malignant ascites

**Figure 2:**
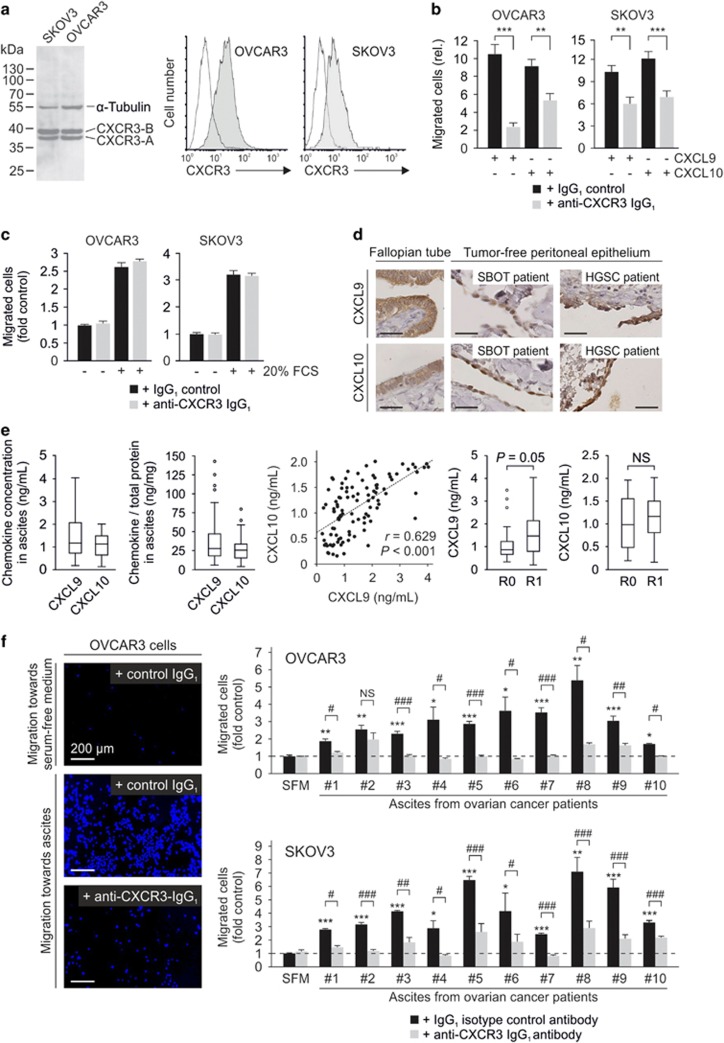
CXCR3 is expressed by ovarian cancer cell lines and mediates their migration toward malignant ascites. (**a**) Western blot analysis showed expression of both CXCR3 splice variants in human OVCAR3 and SKOV3 cells. Flow cytometric analysis revealed CXCR3 surface expression in vital cells of both cell lines. (**b**) CXCL9 and CXCL10 (40 ng/ml) induced migration of human ovarian cancer cell lines, which was inhibited by preincubation of the cells with a monoclonal anti-CXCR3 antibody. (**c**) Unspecific migration of cells toward fetal calf serum was not abrogated by the monoclonal anti-CXCR3 antibody. (**d**) Benign (tumor-free) mesothelium from patients with serous borderline tumors (SBOT) and advanced HGSC expressed CXCL9 and CXCL10. Fallopian tube epithelium was used as a positive control. (**e**) CXCL9 and CXCL10 were detected by ELISA in ascites samples from HGSC patients (*n*=102, left). There was a strong correlation between absolute CXCL9 and CXCL10 concentrations (middle graph). CXCL9 concentrations were significantly higher in suboptimally debulked tumors (R1), that is, with a higher peritoneal tumor burden. This effect was not seen for CXCL10 (right). (**f**) Migration of ovarian cancer cell lines toward malignant ascites from 10 HGSC patients (cell nuclei stained with 4,6-diamidino-2-phenylindole (DAPI), blue). In all cases, ascites-induced migration of OVCAR3 and SKOV3 cells compared with serum-free medium (SFM). Inhibition of CXCR3 with a monoclonal antibody abrogated this migration in most cases compared with an isotype control antibody. NS, not significant. R0, optimally debulked (0 cm). R1, suboptimally debulked (>0 cm). Bars in **d**, 30 μm.

We next explored the function of CXCR3 in ovarian cancer using the two well-established epithelial ovarian cancer (EOC) cell lines OVCAR3 (HGSC) and SKOV3 (clear cell ovarian cancer) as *in vitro* models. Both cell lines express CXCR3 on their cell surface (Figure 2a). Stimulation of the cells with 100 ng/ml of the CXCR3 ligands CXCL9 or CXCL10 did not influence tumor cell proliferation (Supplementary Figure S1). However, both cell lines migrated toward 40 ng/ml CXCL9 or CXCL10 in transwell migration assays (Figure 2b). This migratory activity could be reduced by preincubating the cells with 1 μg/ml of the monoclonal MAB160 anti-CXCR3 antibody 30 min before the migration assay (Figure 2b). This inhibition was specific as the antibody did not block an unspecific migration toward fetal calf serum (Figure 2c).

Next, we looked for the potential source of migratory stimuli that could foster tumor cell migration toward the peritoneal cavity in ovarian cancer patients. Benign mesothelium both from patients with serous borderline tumors and HGSC expressed CXCL9 and CXCL10 (Figure 2d). Both chemokines were also detected by enzyme-linked immunosorbent assay (ELISA) in 102 ascites samples from HGSC patients collected during primary surgery (Figure 2e). Median CXCL9 and CXCL10 concentrations were 1.18 ng/ml (range 0.20–4.03 ng/ml) and 1.14 ng/ml (0.16–2.01 ng/ml), respectively, (27.86 ng/mg total protein (6.20–142.18 ng/mg) for CXCL9 and 25.59 ng/mg total protein (3.88–80.00 ng/mg) for CXCL10). Thus, both chemokine concentrations were within ranges that permit cell migration according to prior reports.^19^ There was a strong correlation between both chemokine concentrations (*r*=0.629; *P*<0.001; Figure 2e). Ascites of patients that could not be optimally debulked during primary surgery, a surrogate for higher peritoneal tumor load, contained significantly higher CXCL9 concentrations than ascites from patients that were optimally debulked (1.47 ng/ml (0.20–4.03 ng/ml) vs 0.86 ng/ml (0.33–3.48 ng/ml), *P*=0.05; Figure 2e). For CXCL10, there was no such difference observed (1.16 ng/ml (0.16–2.01 ng/ml) vs 0.98 ng/ml (0.19–1.96 ng/ml)). The third CXCR3-A ligand CXCL11 had been shown before not to be present in ovarian cancer ascites in detectable amounts.^20^

We next examined the relevance of CXCR3 for directional migration of EOC cells toward malignant ascites, as a surrogate for the intraperitoneal chemotactic milieu. Ten samples from the above cohort were randomly chosen. Clinicopathological characteristics and CXCR3 chemokine concentrations are given in Supplementary Table S2. In transwell migration assays, all samples caused a significant increase in OVCAR3 and SKOV3 cell migration as compared with the random migration seen toward serum-free medium (Figure 2f). Of note, we did not observe a correlation between the absolute chemokine concentration and the extent of tumor cell migration. However, in contrast to the isotype control antibody, inhibition of CXCR3 by the monoclonal MAB160 anti-CXCR3 antibody caused a significant reduction of directional migration in all but one case, mostly down to the level of random migration (Figure 2f). On average, CXCR3 inhibition reduced the ascites-induced migration of OVCAR3 cells by 86.4±7.1% and that of SKOV3 cells by 85.7±6.5%.

### Inhibition of CXCR3 reduces EOC cell migration toward ascites in human ovarian cancer patients *ex vivo*

**Figure 3:**
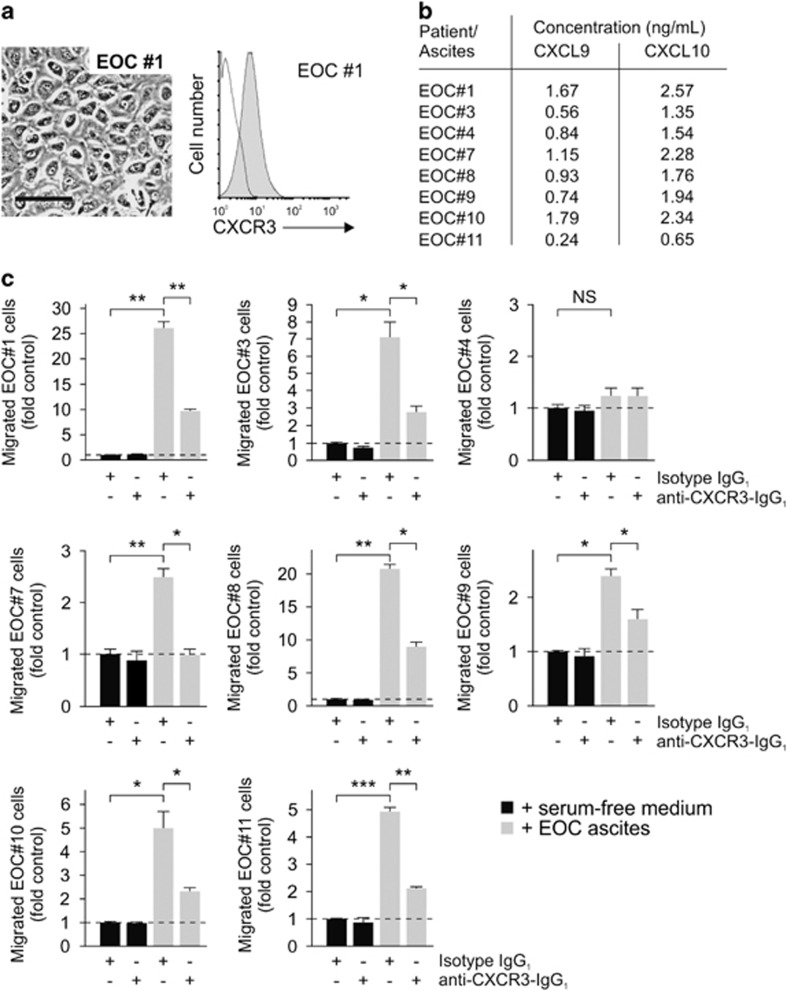
CXCR3 mediates migration of ascites-derived primary cultures of human cancer cells *ex vivo.* Malignant ascites was prospectively collected from 11 EOC patients who underwent paracentesis (Supplementary Table S1). (**a**) Tumor cells were isolated from ascites, characterized and analyzed for CXCR3 surface expression (here shown for EOC#1). (**b**) Absolute CXCL9 and CXCL10 ascites concentrations were determined by ELISA. No tumor cells grew from the ascites of patients #2, #5 and #6. (**c**) Primary tumor cells were subjected to migration assay using the ascites they were isolated from as chemoattractant. Inhibition by the monoclonal anti-CXCR3 antibody MAB160 was tested. Bar in **a**, 100 μm.

To validate our *in vitro* results, we prospectively isolated malignant ascites from 11 EOC patients and generated primary tumor cell cultures (patient characteristics are given in Supplementary Table S1). The ascites of three patients (#2, #5 and #6) did not yield stable primary cell cultures. Using the protocol described by Shepherd *et al.*,^21^ fibroblast contamination was not observed in the remaining eight patients. All ascites-derived tumor cells expressed the CXCR3 receptor detected by flow cytometry (Figure 3a, Supplementary Figure S2). CXCL9 and CXCL10 ascites concentrations were measured by ELISA (Figure 3b). In all but one case (EOC#4), the ascites of origin induced directional migration of the corresponding EOC cells (Figure 3c). In all of these cases, the monoclonal MAB160 anti-CXCR3 antibody significantly inhibited ascites-directed tumor cell migration (Figure 3c).

### Tumor cell expression of CXCR3 is an independent marker of poor outcome in HGSC patients

**Figure 4:**
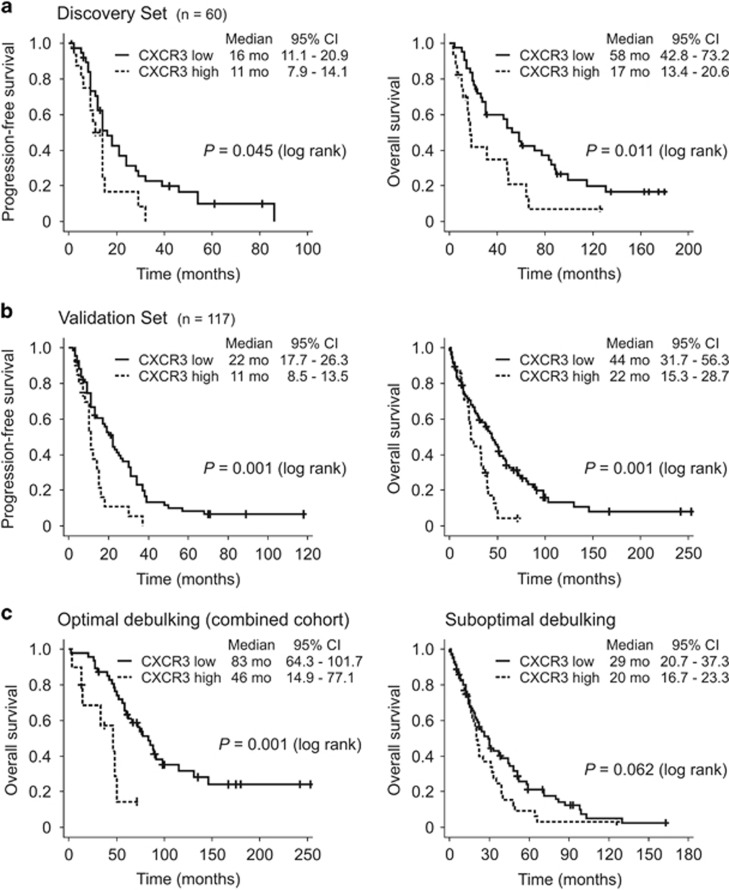
Kaplan–Meier curves showing that high tumor cell CXCR3 (immunohistochemistry score 3+) is associated with shorter PFS (left) and OS (right), both in the discovery set (**a**, *n*=60) and in the validation set (**b**, *n*=117). (**c**) The difference in overall survival was more pronounced in optimally debulked patients (combined cohort). Mo, months.

Finally, we explored the association of tumor cell CXCR3 expression and patient survival in two independent cohorts of HGSC patients.^14^ In the ‘validation set’ (*n*=117, Table 1), tumor cell CXCR3 was scored 0 (7/117, 6%), 1+ (44/117, 38%), 2+ (42/117, 36%) and 3+ (24/117, 21%) (‘discovery set’ see above). In univariate analysis, residual tumor mass after surgery was the strongest indicator of poor PFS and OS in both patient cohorts (Table 2). High expression of CXCR3 in tumor cells (3+) was associated with markedly reduced PFS both in the discovery set (11 vs 16 months, *P*=0.045) and in the validation set (11 vs 22 months, *P*=0.001; Figures 4a and Table 2). With respect to OS, this difference was even more pronounced (discovery set, 17 vs 58 months, *P*=0.011; validation set, 22 vs 44 months, *P*=0.001; Figures 4a and Table 2). Combining the two cohorts, the prognostic effect of high tumor cell CXCR3 on OS was stronger in optimally debulked patients than in those with residual tumor after primary cytoreductive surgery (Figure 4c).

In a multivariate analysis of the combined cohort using postsurgical tumor mass, lymph node involvement, age, and FIGO stage as covariates, high tumor cell CXCR3 expression remained an independent marker for poor PFS and OS in advanced HGSC patients (PFS: hazard ratio (HR) 2.11, 95% confidence interval (CI) 1.30–3.45, *P*=0.003; OS: HR 2.36, 95% CI 1.50–3.71, *P*<0.001; Table 2).

## Discussion

This study in ovarian cancer patients identifies CXCR3 expressed by tumor cells as a novel independent prognostic marker of reduced PFS and OS. In addition, overexpression of CXCR3 at the invasive tumor front and its upregulation in intraabdominal metastases suggest a role for CXCR3 in the selection of tumor cells into peritoneal metastatic niches. Bearing in mind that chemokine receptors primarily facilitate cell migration, one plausible explanation is that CXCR3 mediates tumor cell migration out of the primary tumor. This is supported by our detection of CXCR3 ligands in mesothelial cells and malignant ascites and the reduced tumor cell migration toward this peritoneal milieu upon CXCR3 inhibition.

Chemokine receptors have been previously shown to participate in the metastatic spread of ovarian cancer, most notably the CXCR4/CXCL12 axis.^22^ Overexpression of CXCR4 promotes migration, proliferation and invasion and is associated with poor prognosis in ovarian cancer.^23, 24, 25^ Moreover, inhibition or knock down of CXCR4 resulted in reduced peritoneal dissemination in mouse models of ovarian cancer.^23, 26, 27^ Similarly, overexpression of another chemokine receptor, XCR1, in human ovarian cancer cells leads to increased peritoneal dissemination following intraperitoneal injection of tumor cells into nude mice.^28^ The CX3CR1 chemokine receptor mediates adhesion of ovarian cancer cell to the mesothelial surface via membrane-bound chemokine CX3CL1.^29^ However, in most studies the initial step of tumor dissemination, which is leaving the primary tumor, is skipped by the intraperitoneal injection of tumor cells. Further *in vivo* studies using orthotopic models are warranted to clarify this initial process and to identify feasible target molecules, including the role of CXCR3.

Our finding that tumor cell CXCR3 is associated with poor prognosis in HGSC is in good agreement with prior results in glioma, melanoma, osteosarcoma, colon and breast cancer where it has been functionally linked to lymph node, bone, liver or lung metastasis.^30, 31, 32, 33, 34, 35, 36, 37, 38^ The localization of CXCR3 in tumor cells, endothelial cells and tumor-infiltrating lymphocytes in our HGSC samples matches earlier results in clear cell ovarian cancer.^39^ By using splice variant-specific antibodies, Furuya *et al.*^39^ demonstrated that endothelial cells mainly expressed CXCR3-B, whereas cancer cells mainly expressed CXCR3-A. We detected both splice variants in ovarian cancer cells. Prior studies in several cell types have already identified the CXCR3-A splice variant to be responsible for cell migration.^11^ Our observation that CXCR3 is particularly expressed by tumor cells at the invasive front has been described for other chemokine receptors as well, such as CXCR4 in colorectal and pancreatic cancer.^40, 41^

Our detection of CXCR3 ligands in mesothelial cells of the peritoneal wall and in chemotactically active concentrations in malignant ascites suggests a self-sustaining loop in peritoneal metastasis: inflammatory cytokines such as interferon-γ or tumor necrosis factor-α secreted by the primary tumor either into the circulation or into the peritoneal fluid could induce expression of CXCR3 ligands in mesothelial cells.^42, 43^ This in turn could chemotactically trigger the migration of ovarian cancer cells along the peritoneal cavity via CXCR3. Such mechanisms may in part explain the disappointing therapeutic effects of interferon-γ in the first-line treatment of ovarian cancer,^44^ as well as the pro-metastatic effects of inflammatory cytokines in ovarian cancer models.^45^ Moreover, the involvement of a receptor of lymphocytic chemotaxis could also contribute to the homing of tumor cells predominantly to the ‘milky spots’ of the greater omentum.^46, 47, 48^ These milky spots represent distinct immunologic niches, and it has recently been shown that soluble factors secreted by these milky spots promote ovarian cancer cell migration.^49^ However, the factors involved have not yet been identified, and the expression of CXCR3 ligands by the milky spots of the greater omentum has yet to be proven.

It is important to note that CXCR3 has opposing functions in solid cancers: on the one hand, it mediates the tumor-supressive lymphocytic infiltration of CXCR3-positive immune cells, on the other hand, it is used by tumor cells to proliferate, invade and migrate.^11^ Recently, we have shown that a high intratumoral expression of CXCL9 and CXCL10 is associated with improved overall survival in advanced HGSC.^14^ Although the protective effect of intratumoral CXCR3 ligands is generally attributed to an enhanced tumor-suppressive immune infiltrate, tumor-promoting lymphocytes such as regulatory T cells also use CXCR3 to home into ovarian cancers.^14, 17^ Although the net effect may still be in favor of a tumor-suppressive immune milieu, the results of this study suggest another mechanism that may add to the protective effect of intratumoral CXCL9 and CXCL10: the retention of CXCR3-positive cancer cells in the tumor bed. To further back this hypothesis, a comparison between the intratumoral and the extratumoral chemokine concentrations would be desirable, but this approach is limited because of methodological difficulties in determining exclusively the extracellular chemokine concentrations in tumor tissues. Still, the double-edged role of CXCR3 jeopardizes any CXCR3-targeted therapy apporach; targeting tumor cell CXCR3 in cancer patients may simultaneously limit the CXCR3-mediated lymphocytic tumor infiltration. Nevertheless, the seclusion of the peritoneal cavity as a self-contained therapeutic space may allow such therapeutic concepts.^2^ In the past, intraperitoneal antibody therapies have been already proven practicable in advanced ovarian cancer.^50^ As most EOC patients are diagnosed with metastatic disease, a CXCR3-directed therapy to prevent metastatic spread is only conceivable as part of an adjuvant therapy after primary debulking surgery. This is supported by our finding that CXCR3 overexpression discriminated best between poor and good outcome in patients after complete tumor resection. Further *in vivo* studies are now warranted to elucidate the therapeutic feasibility of such an anti-CXCR3 treatment.

In conclusion, our analyses in primary patient samples indicate that CXCR3 is a driver of tumor cell migration toward the peritoneal environment in human ovarian cancer. Our results advocate further studies assessing CXCR3 as a potential target for ovarian cancer therapy.

## Materials and methods

### Patient characteristics

For the immunohistochemical studies, two independent cohorts of formalin-fixed, paraffin-embedded specimens from 177 patients with advanced HGSC treated at our institution between 1994 and 2010 were used (discovery set, *n*=60; validation set, *n*=117) as described before.^14^ Detailed patient characteristics are given in Table 1. All patients underwent standard debulking surgery followed by platinum-based chemotherapy. Patients receiving neoadjuvant chemotherapy were excluded to study the effects of CXCR3 physiology in its unaffected state. For a subset of 102 samples, matched primary and metastatic lesions, including lymph node, peritoneal and omental metastases, were available for analysis. The study was approved by the Institutional Review Board of the Technical University of Munich (approval 5747/13).

### Cell lines and antibodies

OVCAR3 and SKOV3 (American Type Culture Collection, Manassas, VA, USA) human ovarian cancer cell lines were cultured in a humidified 5% (v/v) CO_2_ atmosphere at 37 °C in Dulbecco’s modified Eagle’s medium supplemented with glutamine, 10% (v/v) fetal calf serum, 10 mM HEPES and 20 μg/ml gentamycin. Cell lines were regularly tested for mycoplasma contamination. OVCAR3 culture medium additionally contained 0.01% (v/v) insulin. Antibodies are as follows: monoclonal mouse IgG_1_ against human CXCR3 (clone 49801, MAB160, R&D Systems, Minneapolis, MN, USA); IgG_1_ isotype control antibody (clone 11711, MAB002, R&D Systems); GAPDH (MAB374, Merck, Darmstadt, Germany); IgG_2a_ against α-tubulin (clone B-7, sc-5286, Santa Cruz Biotechnology, Santa Cruz, CA, USA).

### CXCR3 immunohistochemistry

CXCR3 immunohistochemistry was performed on full slides of 177 primary tumors and 102 corresponding metastases. In all, 2 μm slides were deparaffinized using xylene followed by a graded series of alcohol (100–70%), rehydrated in H_2_O^dist^ and subjected to heat-induced epitope retrieval in citrate buffer (pH 6.0). Endogenous peroxidase activity was blocked by treatment of the sections with 3% (v/v) H_2_O_2_, 20 min, room temperature (RT), followed by endogenous avidin/biotin block and subsequent incubation with goat serum. The sections were then incubated (1 h, RT) with 0.5 μg/ml of anti-CXCR3 monoclonal antibody diluted in green antibody diluent (ZUC025, Zytomed Systems, Berlin, Germany). For detection of the primary antibody-binding reaction, the LSAB-Kit (Zytomed Systems) was used according to the manufacturer’s instruction. Sections were washed thoroughly between incubations, and cell nuclei counterstained with Meyer’s hematoxylin. Histological images were taken using the digital slide scanner NanoZoomer Digital Pathology RS (Hamamatsu, Japan). Normal epithelium from unaffected fallopian tubes was used as a control tissue for each staining. CXCR3 staining in tumor cells was assessed semiquantitatively as absent (0), weak (1+), moderate (2+) or strong (3+). Evaluators were blinded to the clinical data. Staining pattern was membranous and cytoplasmic in all positive cases. In cases with positive staining, all tumor cells were positive, and therefore percentage of positivity was not included in our evaluation. In cases of intratumoral heterogeneity, the staining intensity of the majority of tumor cells was chosen. In cases with strongest staining intensity at the invasive margin, this intensity was recorded regardless of proportion.

### Western blot analysis

Immunoblotting onto nitrocellulose membranes was performed as described.^51, 52^ Tissue extracts from fresh-frozen ovarian cancer tissues were prepared as described.^53^ Primary antibodies were applied as follows: anti-CXCR3 0.67 μg/ml, anti-GAPDH 0.1 μg/ml and anti-α-tubulin 0.2 μg/ml.

### Primary culture of ascites-derived tumor cells from ovarian cancer patients

For the prospective *ex vivo* validation study, we isolated and characterized tumor cells from patients with advanced HGSC undergoing paracentesis using the protocol described by Shepherd *et al.*^21^ Patient characteristics are listed in Supplementary Table S1.

### Flow cytometry

In order to assess CXCR3 cell surface expression, living cells were stained with anti-CXCR3 or IgG_1_ isotype control antibody (20 μg/ml in 0.5% fetal calf serum (w/w), 0.01% NaN_3_ (w/v), 1 h on ice). Subsequently, cells were incubated with Alexa Fluor 488-conjugated antibody A-11001 (2.85 μg/ml in 0.5% (v/v) fetal calf serum, 0.01% NaN_3_ (w/v), 30 min on ice). Dead cells were identified upon simultaneous staining with propidium iodide. Fluorescence intensity as a measure of antibody binding or 7-aminoactinomycin D reaction with cell nuclei was recorded using a FACSCalibur (Becton-Dickinson, Heidelberg, Germany). Histograms were evaluated and plotted using Flowing Software 2 (Version 2.5.1, Turku Center of Biotechnology, Finland).

### Migration assay

Migratory capacity of cancer cells toward chemoattractants and ascites, cell migration was assayed in 24-well modified Boyden chambers with 8.0 μm pore size polycarbonate membranes (6.5 mm Transwell, Corning Inc., Corning, NY, USA). Membranes were first hydrated in 500 μl serum-free culture medium for 1 h. Then, cancer cells were detached, washed and seeded in the upper chamber of the inserts at a density of 5 × 10^4^ cells per well in 500 μl serum-free medium. In all, 500 μl of serum-free medium supplemented with chemoattractant (40 ng/ml human CXCL9, CXCL10 or 0.1% bovine serum albumin (w/v)/phosphate-buffered saline as a control) or 500 μl of ascitic fluid were added to the lower chamber. In the neutralization assay, anti-CXCR3 or IgG_1_ isotype control antibody was added to the cells in the upper chamber in a concentration of 1 μg/ml 30 min before the addition of the chemoattractant to the lower chamber. After 4 h, the media were removed, the membranes washed in phosphate-buffered saline and non-migrated cells scraped off with cotton swabs. Membranes were fixed and stained with 1 μg/ml 4,6-diamidino-2-phenylindole in methanol for 15 min. After washing in phosphate-buffered saline containing Ca^2+^ and Mg^2+^ and high-purity water, the membranes were dried, cut out with a scalpel and sealed on microscope slides with coverslips, using Vectashield mounting medium and nail polish. The migrated cells were visualized and counted in five different fields using the Zeiss Axio Observer A1 fluorescence microscope (Zeiss, Jena, Germany). Migration was normalized against the spontaneous migration seen toward serum-free medium.

### Enzyme-linked immunosorbent assay

Concentrations of CXCR3 ligands CXCL9 and CXCL10 in ascites of ovarian cancer patients, we determined using the Duoset ELISA kits DY392 and DY266 (R&D Systems), respectively, according to the manufacturer’s instructions.

### Statistical analyses

Univariate survival analyses were plotted using the Kaplan–Meier method and analyzed with the log-rank test. For multivariate survival analyses, a Cox proportional hazard model was used. All migration experiments were analyzed using the Mann–Whitney test (SPSS Statistics Software, Version 22.0, SPSS Inc., Chicago, IL, USA). McNemar’s test was used to compare CXCR3 expression in primary tumors vs metastatic lesions. Results are given as mean±s.e.m., if not indicated otherwise. Statistical significance was defined as *, ^#^*P*⩽0.05; **, ^##^*P*⩽0.005; or ***, ^###^*P*⩽0.001.

## Figures and Tables

**Table 1:** Patient demographics and clinicopathologic characteristics of the discovery and validation cohorts

*Characteristic*	*Discovery set (*n=*60)*	*Validation set (*n=*117)*	*All patients (*n=*177)*
Median age at diagnosis (years) (range)	62.5 (35–81)	64 (33–88)	63 (33–88)
⩽65	35 (58)	69 (59)	104 (59)
>65	25 (42)	48 (41)	73 (41)
Median follow-up time PFS (months) (range)	13.5 (1–86)	14.5 (2–118)	14 (1–118)
Median follow-up time OS (months) (range)	31 (3–180)	33 (1–253)	33 (1–253)

*FIGO stage*			
III	47 (78)	80 (68)	127 (72)
IV	13 (22)	37 (32)	50 (28)

*Postsurgical residual tumor mass*			
Optimal (0 cm)	19 (32)	39 (33)	58 (33)
Suboptimal	41 (68)	76 (65)	117 (66)
No data available		2 (2)	2 (1)

*Nodal status*			
Negative (pN0)	14 (23)	38 (33)	52 (29)
Positive (pN1)	35 (58)	67 (57)	102 (58)
No data available	11 (19)	12 (10)	23 (13)

Abbreviations: FIGO, Fédération Internationale de Gynécologie et d'Obstétrique; OS, overall survival; PFS, progression-free survival.

Brackets indicate percentage (%) if not indicated otherwise.

**Table 2:** Univariate and multivariate Cox analyses for clinicopathologic factors and tumor cell CXCR3 expression for progression-free and overall survival in advanced high-grade serous ovarian cancer

*Variable*	*Univariate Cox analysis*				*Multivariate Cox analysis*	
	*Discovery set (*n=*60)*		*Validation set (*n=*117)*		*All patients (*n=*177)*	
	*HR (95% CI)*	P*-value*	*HR (95% CI)*	P*-value*	*HR (95% CI)*	P*-value*
*Progression-free survival*						
CXCR3 expression (high vs low)^a^	1.88 (0.98–3.61)	0.059	2.37 (1.37–4.09)	**0.002**	2.11 (1.30–3.45)	**0.003**
Debulking (suboptimal vs optimal)^b^	4.78 (2.14–10.68)	**<0.001**	2.26 (1.40–3.63)	**0.001**	2.52 (1.64–3.86)	**<0.001**
Nodal status (positive vs negative)	1.02 (0.49–2.13)	0.962	1.06 (0.65–1.72)	0.822	0.97 (0.63–1.50)	0.894
Age	1.00 (0.97–1.03)	0.985	1.03 (1.01–1.05)	**0.015**	1.01 (0.99–1.03)	0.491
FIGO stage (IV vs III)	0.81 (0.40–1.65)	0.565	1.67 (1.02–2.73)	**0.043**	1.28 (0.82–2.01)	0.277
						
*Overall survival*						
CXCR3 expression (high vs low)	2.23 (1.18–4.23)	**0.014**	2.16 (1.32–3.52)	**0.002**	2.36 (1.50–3.71)	**<0.001**
Debulking (suboptimal vs optimal)	3.33 (1.68–6.58)	**0.001**	3.17 (1.99–5.07)	**<0.001**	3.23 (2.10–4.97)	**<0.001**
Nodal status (positive vs negative)	1.11 (0.52–2.38)	0.776	1.15 (0.74–1.78)	0.539	0.80 (0.54–1.19)	0.266
Age	1.00 (0.97–1.02)	0.695	1.03 (1.01–1.05)	**0.006**	1.02 (1.00–1.04)	0.078
FIGO stage (IV vs III)	0.85 (0.41–1.76)	0.659	2.17 (1.41–3.33)	**<0.001**	2.16 (1.42–3.30)	**<0.001**

Abbreviations: CI, confidence interval; FIGO, Fédération Internationale de Gynécologie et d'Obstétrique; HR, hazard ratio.

a High=immunohistochemistry score 3+, low=immunohistochemistry scores 0–2+

.

b Optimal=0 cm, suboptimal >0 cm.

Bold entries indicate statistically significant *P*<0.05.
