# Supplementary material for: CXCR3 mediates ascites-directed tumor cell migration and predicts poor outcome in ovarian cancer patients
Source: Oncogenesis. 2017 May 15;6(5):e331–. doi: 10.1038/oncsis.2017.29 (PMC5523062; doi:10.1038/oncsis.2017.29)
Supplement: Supplementary Table 1 [file oncsis201729x1.docx]

**Supplementary Table S1.** Clinicopathological characteristics of ovarian cancer patients whose ascites was the source of primary tumor cell lines. HGSC, high-grade serous cancer. LGSC, low-grade serous cancer. EOC, epithelial ovarian cancer (not further specified).

| EOC  cell line | Age (yr) | Histologic subtype | FIGO stage | Nodal status (pN) | Postsurgial tumor rest | Comment |
| --- | --- | --- | --- | --- | --- | --- |
| #1 | 38 | HGSC | III | 1 | No |  |
| #2 | 63 | LGSC | IC | Unknown | No | Not grown |
| #3 | 69 | HGSC | III | 1 | No |  |
| #4 | 73 | HGSC | III | Unknown | Yes |  |
| #5 | 71 | HGSC | III | 1 | No | Not grown |
| #6 | 71 | HGSC | III | 1 | No | Not grown |
| #7 | 93 | EOC | III | Unknown | Yes |  |
| #8 | 71 | HGSC | IV | 0 | Yes |  |
| #9 | 81 | EOC | III | 0 | Yes |  |
| #10 | 72 | HGSC | III | 1 | Yes |  |
| #11 | 76 | HGSC | III | 1 | No |  |
