# Supplementary material for: CXCR3 mediates ascites-directed tumor cell migration and predicts poor outcome in ovarian cancer patients
Source: Oncogenesis. 2017 May 15;6(5):e331–. doi: 10.1038/oncsis.2017.29 (PMC5523062; doi:10.1038/oncsis.2017.29)
Supplement: Supplementary Table 2 [file oncsis201729x2.docx]

**Supplementary Table S2.** Clinicopathological characteristics of the ascites samples used for the *in vitro* migration assay experiments.

| Sample | Age  (yr) | Histologic subtype | FIGO stage | Nodal status (pN) | Residual tumor after surgery | Ascites concentration | |
| --- | --- | --- | --- | --- | --- | --- | --- |
|  |  |  |  |  |  | CXCL9  (ng/mL) | CXCL10  (ng/mL) |
| #1 | 68 | HGSC | III | unknown | yes | 0.29 | 0.13 |
| #2 | 62 | HGSC | IV | 1 | yes | 0.25 | 0.19 |
| #3 | 55 | HGSC | III | 0 | no | 1.31 | 0.83 |
| #4 | 51 | HGSC | IV | 1 | yes | 4.03 | 1.90 |
| #5 | 76 | HGSC | III | 1 | no | 3.56 | 1.30 |
| #6 | 48 | HGSC | IC | unknown | no | 3.42 | 1.80 |
| #7 | 70 | HGSC | III | 1 | yes | 2.66 | 1.71 |
| #8 | 78 | HGSC | III | unknown | yes | 3.63 | 1.77 |
| #9 | 68 | HGSC | IV | 1 | yes | 2.62 | 1.76 |
| #10 | 65 | HGSC | III | 1 | yes | 2.89 | 1.77 |
