# Supplementary figures and images for: CXCR3 mediates ascites-directed tumor cell migration and predicts poor outcome in ovarian cancer patients
Source: Oncogenesis. 2017 May 15;6(5):e331–. doi: 10.1038/oncsis.2017.29 (PMC5523062; doi:10.1038/oncsis.2017.29)

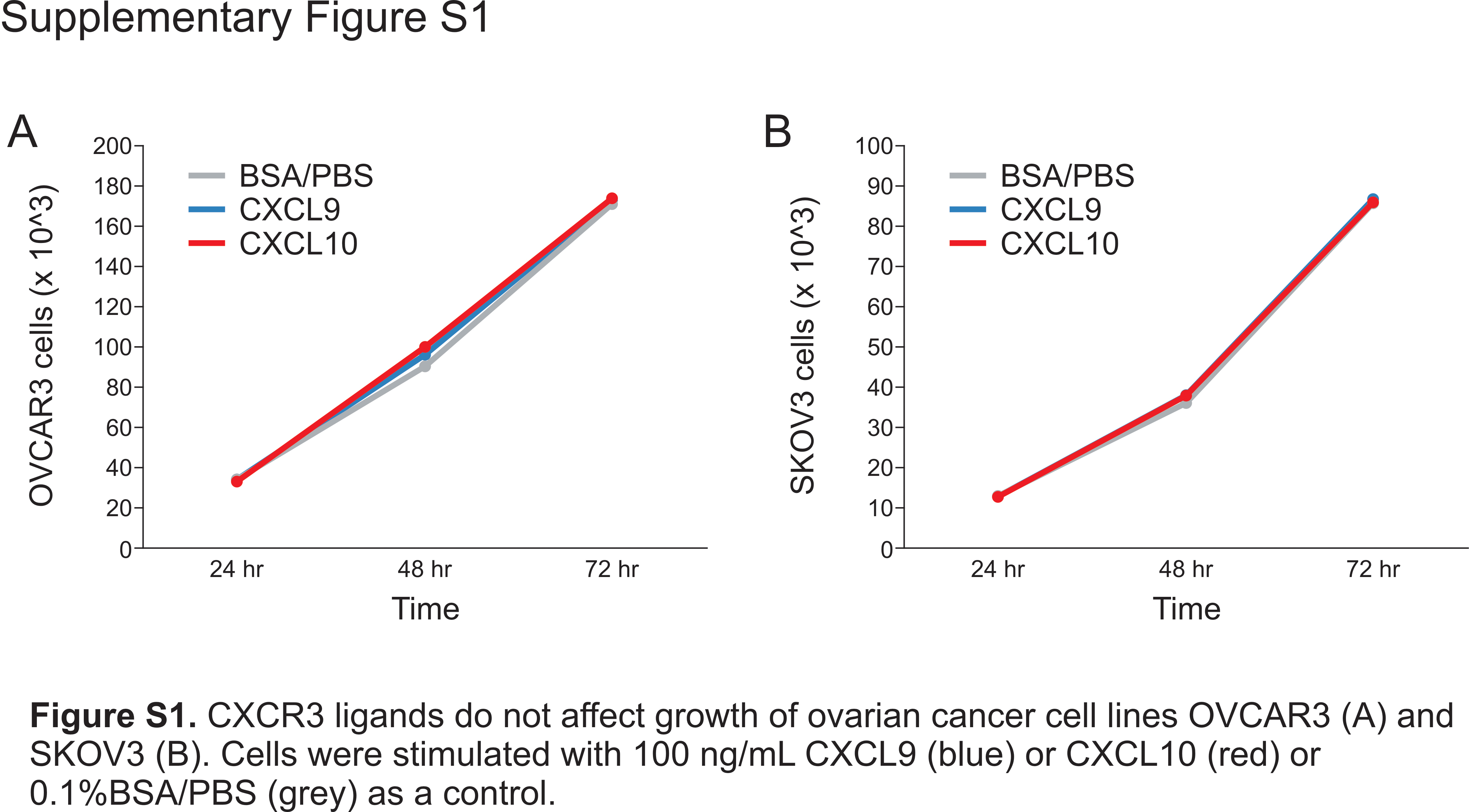

Supplement: Supplementary Figure 1 [file oncsis201729x3.tif]

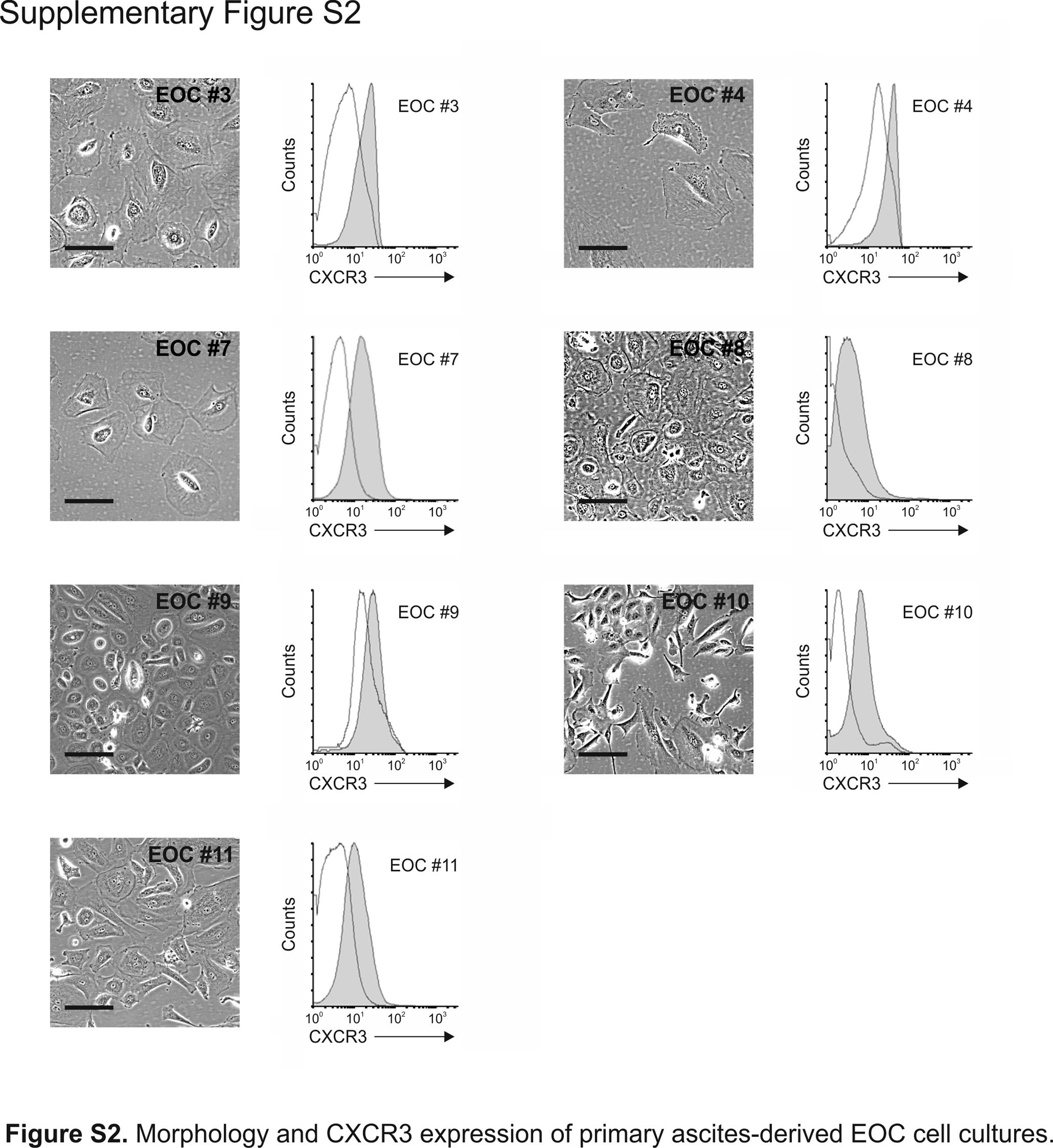

Supplement: Supplementary Figure 2 [file oncsis201729x4.tif]

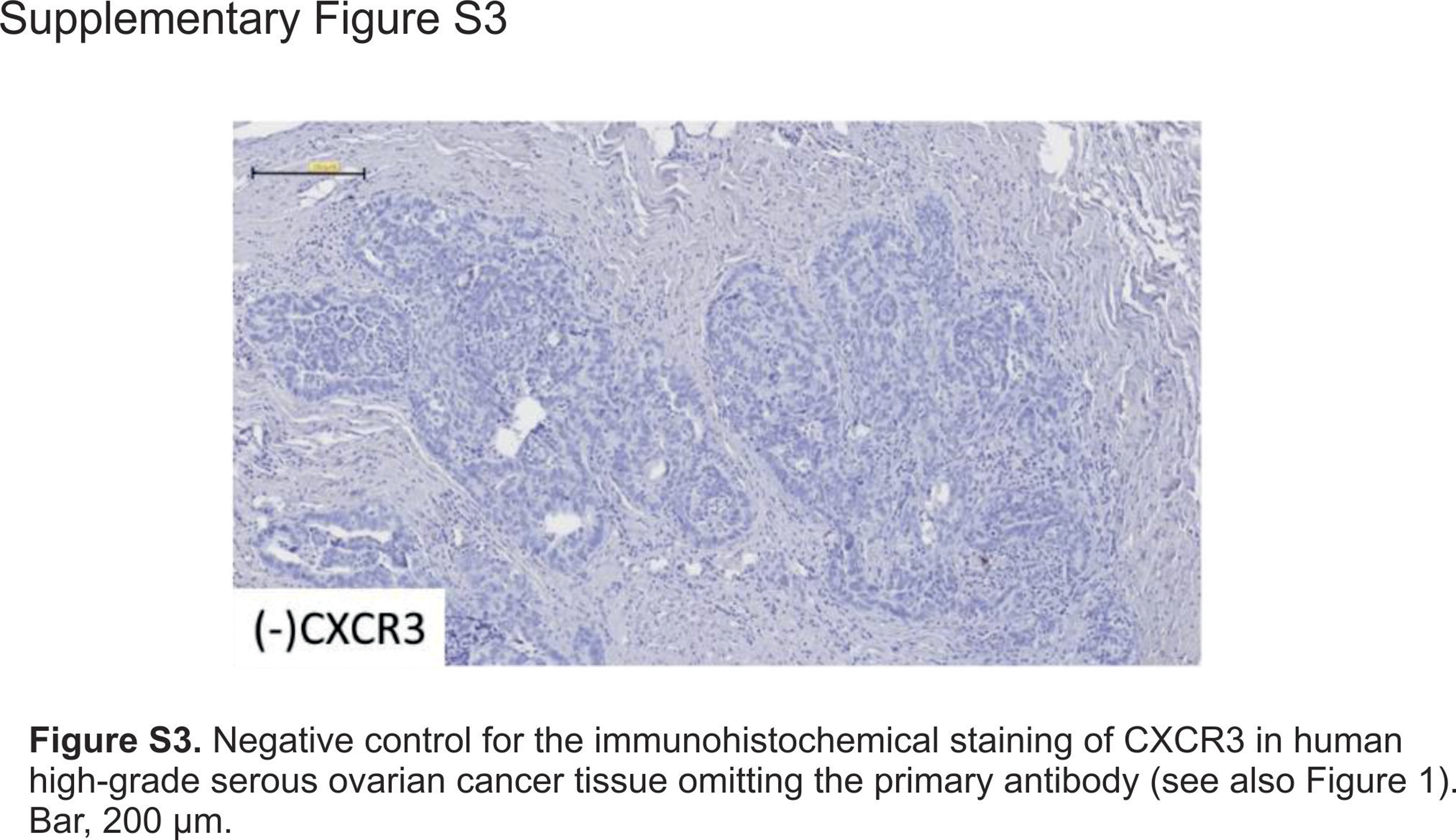

Supplement: Supplementary Figure 3 [file oncsis201729x5.tif]
